# Contribution of social activity participation to the relationship between sensory impairment, physical performance and cognitive decline: a longitudinal study in China

**DOI:** 10.3389/fnagi.2024.1498354

**Published:** 2024-12-11

**Authors:** Lei Lei, Yinuo Zhou, Lizhen Ye, Yanfang Yang

**Affiliations:** ^1^Department of Epidemiology and Biostatistics, West China School of Public Health and West China Fourth Hospital, Sichuan University, Chengdu, China; ^2^Chengdu Shuangliu District Center for Disease Control and Prevention, Chengdu, Sichuan, China; ^3^Department of Public Health, Erasmus MC, University Medical Center, Rotterdam, Netherlands

**Keywords:** cognitive function, sensory impairment, physical performance, social activity participation, mediation analyses

## Abstract

**Objectives:**

This study examined the impact of sensory impairment (hearing and/or vision) combined with poor physical performance on cognitive decline and assessed whether social activity participation mediated this relationship.

**Methods:**

Multilevel models were used to explore the relationships between sensory impairment, physical performance, their combination, and cognitive decline. A multilevel mediation model analyzed the mediating effect of social activity participation. The study included 10,149 adults aged 45 and older (mean age 53.84) from five waves (2011–2020) of the China Health and Retirement Longitudinal Study (CHARLS). The exposure variable, body function (BF), was categorized into six groups based on sensory impairment and physical performance. Cognitive function was measured using an adapted Chinese Mini-Mental State Examination (MMSE).

**Results:**

Compared to BF group 1 (normal physical performance without sensory impairment), individuals in BF group 4 (low physical performance without sensory impairment) (β = −0.670, *P* < 0.001), BF group 5 (low physical performance with single sensory impairment) (β = −1.029, *P* < 0.001), and BF group 6 (low physical performance with dual sensory impairment) (β = −1.630, *P* < 0.001) showed worse cognitive function. Only BF group 4 (β = −0.670, *P* < 0.001) had a faster cognitive decline. Mediation analysis revealed a significant indirect effect of social activity participation on cognitive function in BF group 4 (β = −0.03, *P* < 0.01, mediation proportion: 4.32%).

**Conclusion:**

The combined effect of sensory impairment and low physical performance on cognitive decline may be accumulative, and social activity participation could mitigate this effect. Prioritizing social activity participation is crucial for health professionals and policymakers.

## 1 Introduction

China is experiencing both a growing number of individuals reaching old age and an accelerated aging rate (World Health Organization, 2022). It is projected that by 2040, approximately 28% of the Chinese population will be aged 60 years and above (Bao et al., 2022). Consequently, the increasing burden of disease and healthcare demands within society will accompany this large older population, as aging naturally entails irreversible deterioration of physical and mental capacities over time, leading to various age-related diseases (Titorenko, 2019).

Alzheimer’s disease and related dementias (ADRD) are among the age-related diseases and became the fifth leading cause of death in China in 2019 (Ren et al., 2022). Throughout the progression of dementia, cognitive decline acts as a potential early indicator (Mitchell, 2008; Centers for Disease Control and Prevention, 2011; Kohler et al., 2013). Cognitive decline which characterized by impairments in memory, processing speed, learning new things and concentrating, is a common complication of dementia (Kohler et al., 2013). Research has shown that most individuals with Alzheimer’s disease have cognitive impairment (Kverno, 2022). Additionally, cognitive decline is a risk factor for dementia, and individuals with cognitive impairment have a higher probability of developing Alzheimer’s disease. Because there is no definitive cure for dementia or cognitive decline, identifying modifiable risk factors is crucial to preventing adverse health consequences and the onset of dementia (Northey et al., 2018).

Previous studies have demonstrated that physical performance predict poorer cognitive status (Veronese et al., 2016; Stijntjes et al., 2017). This may be because physical activity in late life serves as a protective factor for brain health (Ai et al., 2024), such as gray matter volume, white matter integrity, task-related activity, or resting-state functional connectivity. Additionally, aerobic exercise has been shown to improve cerebral blood flow and may promote the development of new neural cells, enhance plasticity, and support cell survival through increased levels of BDNF (brain-derived neurotrophic factor) (Leger et al., 2024; Zhang et al., 2024). Additionally, research conducted by Schubert (Schubert et al., 2019) and Christensen (Christensen et al., 2019) et al., has shown that sensory impairment (hearing or vision) was associated with a higher risk of cognitive impairment. Sensory impairments, such as vision or hearing loss, can reduce an individual’s ability to acquire information from the environment, which in turn may affect their understanding and response to their surroundings. When individuals face sensory impairments, the cognitive load required to process information increases. This additional cognitive burden may lead to fatigue and decreased attention, thereby impacting cognitive performance. While interventions such as training programs (Concha-Cisternas et al., 2023) or assistive tools (Spierer et al., 2016; Babajanian and Gurgel, 2022) can partially improve physical performance or sensory impairment to prevent cognitive decline, they require financial costs (Marx et al., 2019) and professional technical guidance which are burdensome for older adults in developing country like China. Thus, this study aimed to identify a potential mediator between sensory impairment, poor physical performance and cognitive decline to find modifiable risk factors which it sought to provide suggestions for daily actions that older adults with poor physical performance or sensory impairments can easily adopt to combat cognitive decline.

Recent research has shown older individuals with sensory impairment (hearing and vision) (Bourque et al., 2007) engaged in fewer social activities, which related cognitive function (Fu et al., 2018). Previous studies have also indicated that the association between hearing loss and cognitive impairment may be mediated by social isolation or loneliness (Maharani et al., 2019), and poor physical performance was reported to be associated with higher risk of social isolation (Del Pozo Cruz et al., 2021). This may be due to physical impairments (such as mobility issues and balance problems) and sensory impairments (such as hearing or vision loss), which can create obstacles for individuals in participating in social activities, such as difficulties in going out, conversing with others, or understanding information in the environment. These participation challenges may lead to a reduction in social activities, thereby increasing feelings of isolation. Thus, social activity participation (SAP) could potentially serve as a mediator between sensory impairment, poor physical performance and cognitive decline. However, available studies rarely address the mediation of SAP between them. Furthermore, studies have reported that vision or hearing impairments are directly associated with decline in physical characteristics, such as muscle strength, physical performance, and cardiorespiratory fitness (Kim et al., 2021; Miyata et al., 2021; Kong et al., 2023). This suggests that when analyzing the impact of sensory impairment and poor physical performance on cognitive decline, it is crucial to consider whether they coexist or occur separately. However, existing research have primarily examined these factors in isolation.

Therefore, based on the aforementioned evidence, we propose two hypotheses. Firstly, we hypothesize that individuals with combined sensory impairment (hearing and/or vision) and poor physical performance would have a higher risk of experiencing cognitive decline compared to those with either condition alone. Secondly, we postulate that SAP plays a significant role in the association between sensory impairment (hearing and/or vision), low physical performance, their combination, and cognitive impairment. Considering previous studies have provided limited insights into these aspects.

This study seeks to explore whether participation in social activities mediates the association between physical and sensory impairments and cognitive decline among a large cohort of older Chinese adults, offering insights into modifiable, accessible factors that could help mitigate cognitive decline in aging populations facing similar challenges.

## 2 Materials and methods

### 2.1 Study design and participants

The data for this study were drawn from the five waves of the China Health and Retirement Longitudinal Study (CHARLS) conducted from 2011 to 2020. This nationally representative longitudinal survey targets Chinese individuals aged 45 and older and their spouses and is authorized by the Ethics Review Committee of Peking University (IRB00001052-11014). To ensure sample representativeness, multistage probability sampling was conducted across 150 counties and 450 communities in 28 provinces. The baseline survey (wave 1) was conducted in 2011 while follow-up surveys were conducted in 2013 (wave 2), 2015 (wave 3), and 2018 (wave 4). The response rates for each wave were 80.5% in 2011, 82.6% in 2013, 82.1% in 2015, 83.8% in 2018 and 84.3% in 2020, respectively (Zhao et al., 2014). For a comprehensive understanding of the survey design and procedures employed in the CHARLS, detailed explanations can be found in the cohort profile literature (Zhao et al., 2014).

**FIGURE 1:**
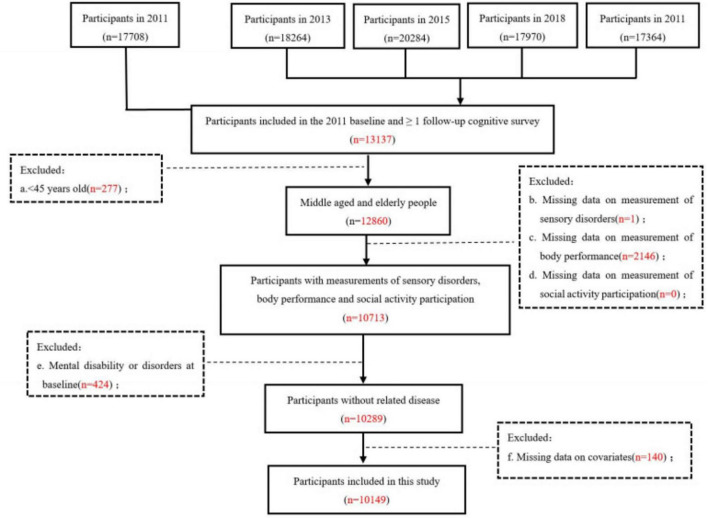
The flow chart of study population.

This study included individuals who had undergone baseline cognitive function assessments in 2011 and at least one follow-up cognitive function assessments in 2013, 2015, 2018, and 2020. Participants were excluded if they met any of the following criteria: (1) younger than 45 years (at baseline survey for each participant); (2) lack of information on independent variables and mediators at baseline; (3) presence of doctor-diagnosed brain damage, intellectual disabilities, psychiatric problems, or memory-related disorders at baseline; and (4) missing information on covariate at baseline. In addition, samples with missing data on key variables were excluded. A total of 10,149 subjects were included in the analysis. The recruitment process of this study was illustrated in Figure 1.

Mediation analyses, which have been increasingly utilized in researches (Hiratsuka et al., 2020; Liu et al., 2022), statistically examine the mediators that intervene between an exposure variable and outcome while considering of covariates.

### 2.2 Measurements

#### 2.2.1 Cognitive function

Based on previous research, cognitive function (CF) was assessed using a methodology adapted from the American Health and Retirement Study (HRS) (Hu et al., 2022; Yao et al., 2022), focusing on four dimensions: orientation, memory, computation, and drawing (Crimmins et al., 2011). The overall cognitive function score was determined by summing the scores from the four dimensions, resulting in a total score ranging from 0 to 31. A higher score on the CF test indicates a better CF.

#### 2.2.2 Physical performance and sensory impairment

Physical performance was assessed using the Short Physical Performance Battery (SPPB) (Guralnik et al., 1994). A summary physical performance score was calculated by summing the scores from three tests (the standing balance test, the five-time chair stand test (5CST) and the gait speed test), resulting in a range of 0–12 points, with 4 points allocated to each test (Zhong et al., 2021). Based on previous studies (Welch et al., 2021; Li et al., 2023; He et al., 2024), normal physical performance is defined as SPPB > 9, while low physical performance is defined as either SPPB ≤ 9 or 5CST ≥ 12 s. A lower score on the SPPB test indicates a worse physical performance.

Sensory impairment was evaluated using the question, “Do you have any difficulties with your vision or hearing?” In this study, single sensory impairment was defined as the presence of vision or hearing problems and dual sensory impairment was defined as the presence of both, while the absence of such problems indicated no sensory impairment.

Participants were categorized into six groups based on their physical performance and the presence of sensory impairment. These groups were named according to their body function (BF) level as follows: (1) Body function group 1 (BF1): Normal physical performance without sensory impairment; (2) Body function group 2 (BF2): Normal physical performance with single sensory impairment; (3) Body function group 3 (BF3): Normal physical performance with dual sensory impairment; (4) Body function group 4 (BF4): Low physical performance without sensory impairment; (5) Body function group 5 (BF5): Low physical performance with single sensory impairment; (6) Body function group 6 (BF6): Low physical performance with dual sensory impairment.

#### 2.2.3 Social activity participation

Social activity participation (SAP) was assessed by asking participants how often they had engaged in six social activities within the past month. The six social activities include: (1) Interacted with friends; (2) Played Ma-jong, played chess, played cards, or went to community club; (3) Provided help to family, friends, or neighbors who do not live with you and who did not pay you for the help; (4) Went to a sport, social, or other kind of club; (5) Took part in a community-related organization; (6) Done voluntary or charity work. Referring to previous research (Fang et al., 2022), we assigned possible responses as: 0 = no participation, 1 = every week or less regularly, and 2 = almost daily. The total scores of SAP measuring by summing up the responses for each of the six activities ranged from 0 to 12, with the higher scores denoting the higher levels of SAP. Cronbach alpha of the social participation measurement was 0.66, indicating the reliability was acceptable (Guilford, 1956).

#### 2.2.4 Covariates

Based on the literature, the covariates of our analysis included demographic characteristics, health status-related variables, health behaviors, socioeconomic level and time variables.

Regarding demographic characteristics, we included age (at baseline), gender (male/female), geographic area of residency (rural/urban), and education level (no formal education, basic literacy or elementary school education, middle or high school, and above).

Health status-related variables were assessed in five aspects, including physical comorbidity, presence of pain (yes/no), instrumental activity of daily living (IADLs) (impaired/not impaired), body mass index (BMI), and depressive symptoms (yes/no). Physical comorbidity was categorized into three categories (0, 1–2 and ≥ 3) (Xu et al., 2022) based on the number of self-reported chronic diseases diagnosis by a physician. Depressive symptoms were assessed using the 10-item Center for Epidemiologic Studies Depression (CESD-10) scale (Andresen et al., 1994). The total CESD-10 score ranged from 0 to 30. Participants with a CESD-10 score above 10 points were classified into the depressive group (Björgvinsson et al., 2013).

This study examined two health behaviors, namely smoking (yes/no) and alcohol intake (yes/no).

Consistent with previous studies (Lei et al., 2014), we calculated the average annual household expenditure per capita to measure the household resources. To capture the non-linear relationship between income and outcome variables, the average annual household expenditure was log-transformed in the analysis.

The time variable was defined as the duration between the baseline and follow-up cognitive assessments for each participant.

Additional details of covariates are available in Supplementary Variable Information and Supplementary Table 1.

Among all the variables mentioned above, the BF was regarded as the exposure variable, while every assessment of CF served as the outcome, and SAP was treated as the mediator.

### 2.3 Statistical analysis

Descriptive statistics were utilized to describe the characteristics of respondents. Continuous variables were presented as mean and standard deviation (SD), while categorical variables were reported as frequency (*n*) and percentage (%). Baseline characteristics were evaluated by chi-square test for categorical variables and one-way analysis of variance (ANOVA) for continuous variables.

Multilevel models were used to assess the relationships between body function (BF) and CF. In this study. The data structure was that up to five waves of repeat measurement data (level 1) were nested within 10,149 individuals (level 2) which means the data were not independently observed. And multilevel models considering data aggregation were applicable to handle dependently and unbalanced data (Diggle and Kenward, 1994; Courgeau, 1997).

We estimated three multilevel models for all respondents finally. Model 1: adjusted model containing time, BF and part of the covariates (age, gender and geographic residence). Model 2: adjusted model containing time, BF and all of the covariates (age, geographic residence, education, physical comorbidities, feeling pain, IADLs, depressive symptoms, smoking, alcohol intake, BMI and household expenditure per capita). Model 3: add the interacting term of BF and time to Model 2. In this study, we incorporated an interaction term between exposure and time. This allows us to identify differences in time effects and reveal dynamic relationships, rather than solely analyzing single time points or overall effects, thereby enhancing the explanatory power of the model. Then we constructed a multilevel mediation model (Model 4) to estimate mediation of SAP between BF and CF for all respondents. Model 4: multilevel mediation model containing BF as exposure, SAP as mediator and CF as outcome with all covariates and time adjusted.

This study supplemented the analysis for comparing baseline characteristics between participants included and not included in Supplementary Table 2.

Data cleaning, descriptive analyses and mediation analyses were conducted using R v4.2.1 and mediation analyses was performed with “mediation” package (Tingley et al., 2014). The “mediation” package supports causal mediation analysis of multilevel data via the “lmer” and “glmer” functions in the “lme4” package (Tingley et al., 2014). *P*-value of < 0.05 was considered statistically significant.

## 3 Results

Table 1 presented the descriptive statistics of groups based on body function level. A total of 10,149 participants were included in the final analyses, with 6,268, 572, 64, 2,721, 420, and 94 for BF group 1–6, respectively. These groups exhibited significant differences in all covariates and baseline cognitive function. The mean age of all participants was 58.34 ± 8.80, and the baseline cognitive function score was 14.60 ± 5.52. The cognitive function score for groups BF1 to BF6 ranged from 15.56 ± 5.00 to 10.39 ± 5.23, respectively. Compared to BF1 group, the other groups with varying degrees of physical performance and sensory impairments exhibited lower cognitive function score.

**TABLE 1:** Characteristics of study population.

Characteristics	Overall (*n* = 10,149)	Body function level						
		**BF1 (*n* = 6,282)**	**BF2 (*n* = 572)**	**BF3 (*n* = 64)**	**BF4 (*n* = 2,717)**	**BF5 (*n* = 420)**	**BF6 (*n* = 94)**	***P*-value**
Cognitive function, mean ± SD	14.60 ± 5.52	15.56 ± 5.00	13.63 ± 4.85	12.84 ± 5.48	13.23 ± 5.34	11.55 ± 5.04	10.39 ± 5.23	< 0.001
Social activity participation, mean ± SD	1.01 ± 1.24	1.09 ± 1.30	0.79 ± 1.16	0.64 ± 0.97	0.91 ± 1.15	0.84 ± 1.07	0.97 ± 1.25	< 0.001
BMI, mean ± SD	23.61 ± 3.93	23.72 ± 3.88	23.09 ± 3.47	23.05 ± 4.00	23.61 ± 4.08	23.04 ± 3.87	22.53 ± 4.60	< 0.001
Age (years at baseline), mean ± SD	58.34 ± 8.80	56.20 ± 7.87	59.45 ± 8.28	61.22 ± 8.30	61.70 ± 9.06	64.73 ± 9.47	67.23 ± 9.47	< 0.001
Gender, *n* (%)								< 0.001
Male	4,885 (48.1)	3,272 (52.1)	314 (54.9)	43 (67.2)	1,034 (38.1)	182 (43.3)	40 (42.6)	
Female	5,264 (51.9)	3,010 (47.9)	258 (45.1)	21 (32.8)	1,683 (61.9)	238 (56.7)	54 (57.4)	
Geographic area, *n* (%)								< 0.001
Rural	878 (8.7)	614 (9.8)	34 (5.9)	4 (6.2)	206 (7.6)	19 (4.5)	1 (1.1)	
Urban	9,271 (91.3)	5,668 (90.2)	538 (94.1)	60 (93.8)	2,511 (92.4)	401 (95.5)	93 (98.9)	
Education, *n* (%)								< 0.001
No formal education	2,537 (25.0)	1,171 (18.6)	166 (29.0)	21 (32.8)	946 (34.8)	181 (43.1)	52 (55.3)	
Basic literacy or elementary school education	4,227 (41.6)	2,568 (40.9)	267 (46.7)	33 (51.6)	1,145 (42.1)	183 (43.6)	31 (33.0)	
Middle or high school	3,207 (31.6)	2,402 (38.2)	134 (23.4)	8 (12.5)	600 (22.1)	54 (12.9)	9 (9.6)	
Above	178 (1.8)	141 (2.2)	5 (0.9)	2 (3.1)	26 (1.0)	2 (0.5)	2 (2.1)	
The number of physical comorbidities, *n* (%)								< 0.001
0	3,396 (33.5)	2,324 (37.0)	153 (26.7)	11 (17.2)	802 (29.5)	89 (21.2)	17 (18.1)	
1–2	3,102 (30.6)	1,968 (31.3)	169 (29.5)	15 (23.4)	813 (29.9)	113 (26.9)	24 (25.5)	
≥ 3	3,651 (36.0)	1,990 (31.7)	250 (43.7)	38 (59.4)	1,102 (40.6)	218 (51.9)	53 (56.4)	
Feeling pain, *n* (%)								< 0.001
No	6,914 (68.1)	4,630 (73.7)	342 (59.8)	30 (46.9)	1,657 (61.0)	220 (52.4)	35 (37.2)	
Yes	3,235 (31.9)	1,652 (26.3)	230 (40.2)	34 (53.1)	1,060 (39.0)	200 (47.6)	59 (62.8)	
IADLs, *n* (%)								< 0.001
No	8,452 (83.3)	5,566 (88.6)	431 (75.3)	46 (71.9)	2,098 (77.2)	261 (62.1)	50 (53.2)	
Yes	1,697 (16.7)	716 (11.4)	141 (24.7)	18 (28.1)	619 (22.8)	159 (37.9)	44 (46.8)	
Depressive symptoms, *n* (%)								< 0.001
No	6,240 (61.5)	4,180 (66.5)	325 (56.8)	22 (34.4)	1,475 (54.3)	201 (47.9)	37 (39.4)	
Yes	3,909 (38.5)	2,102 (33.5)	247 (43.2)	42 (65.6)	1,242 (45.7)	219 (52.1)	57 (60.6)	
Smoke, *n* (%)								< 0.001
No	6,121 (60.3)	3,653 (58.2)	314 (54.9)	25 (39.1)	1,822 (67.1)	254 (60.5)	53 (56.4)	
Yes	4,028 (39.7)	2,629 (41.8)	258 (45.1)	39 (60.9)	895 (32.9)	166 (39.5)	41 (43.6)	
Drink, *n* (%)								< 0.001
No	6,701 (66.0)	3,939 (62.7)	359 (62.8)	36 (56.2)	2,004 (73.8)	301 (71.7)	62 (66.0)	
Yes	3,448 (34.0)	2,343 (37.3)	213 (37.2)	28 (43.8)	713 (26.2)	119 (28.3)	32 (34.0)	
Household expenditure per capita (log), mean ± SD	7.31 ± 1.19	7.39 ± 1.16	7.17 ± 1.16	7.35 ± 1.26	7.21 ± 1.22	6.98 ± 1.23	6.97 ± 1.32	< 0.001

(1) All the variable were measured at baseline interview. (2) The *P*-value in continuous variables were tested using one-way analysis of variance (ANOVA) and in categorical variables using chi-square tests. (3) SD, standard deviation; BMI, body mass index; IADLs, instrumental activity of daily living; BF, body function (Body function groups were classified by whether individuals suffered from sensory disorders or poor physical performance which mentioned in Method of this study).

Table 2 presented results of the multilevel growth model fit for Models 1–3. In Model 1, both time and BF were significantly associated with CF after controlling for age, gender and geographic residence. On average, scores for CF have decreased by 0.115 units per year. In comparison to BF group 1, the scores of CF were lower among participants with single/dual sensory impairment, low physical performance or their combination, by 1.154, 2.391, 1.366, 2.491 and 3.541 for BF group 2–6, respectively. After adjusting for all covariates in Model 2, the time term remained significant (β = −0.116, *P* < 0.001), indicating a negative effect on cognitive function over time. In contrast to BF group 1, individuals of BF group 4 (β = −0.670, *P* < 0.001), BF group 5 (β = −1.029, *P* < 0.001), and BF group 6 (β = −1.630, *P* < 0.001) performed worse on CF. Model 3 examined the differences in the rates of cognitive decline between BF groups by adding the interacting terms of BF and time to Model 2. Compared with BF group 1, only participants of BF group 4 (β = −0.670, *P* < 0.001) showed a faster rate of cognitive decline after controlling for all covariates. The results of Maximum likelihood values for Models 1–3 indicated that Models 2–3 with lower −2log-likelihood values fitted better than Model 1. The −2 log-likelihood is a statistical measure that indicates the goodness of fit of a model. It is calculated as the negative two times the log-likelihood value, with lower values suggesting a better fit to the observed data.

**TABLE 2:** The association between body function and cognitive function.

	Model 1	Model 2	Model 3
**Fixed effects**			
Intercept	21.181 (20.852 to 20.510)***	9.151 (8.423 to 9.75)***	9.103 (8.379 to 9.827)***
**Body function (ref = BF group 1)**			
BF group 2	−1.154 (−1.530 to −0.778)**	−0.285 (−0.589 to 0.021)	−0.315 (−0.668 to 0.386)
BF group 3	−2.391 (−3.472 to −1.310)**	−0.866 (−1.731 to 0.020)	−0.745 (−1.762 to 0.272)
BF group 4	−1.366 (−1.572 to −1.160)***	−0.670 (−0.838 to −0.502)***	−0.499 (−0.691 to −0.305)***
BF group 5	−2.491 (−2.935 to −2.048)***	−1.029 (−1.392 to −0.666)***	−1.090 (−1.507 to −0.673)***
BF group 6	−3.541 (−4.450 to −2.631)***	−1.630 (−2.373 to −0.887)***	−1.389 (−2.230 to −0.538)**
Time (years since baseline)	−0.115 (−0.125 to −0.104)***	−0.116 (−0.126 to −0.105)***	−0.105 (−0.118 to −0.092)***
**Age (ref = 45–54, at baseline)**			
55–64	−1.890 (−2.087 to −1.694)***	−0.421 (−0.588 to −0.255)***	−0.420 (−0.587 to −0.254)***
≥ 65	−3.832 (−4.069 to −3.595)***	−1.756 (−1.961 to −1.551)***	−1.760 (−1.965 to −1.555)***
**Gender (ref = male)**			
Female	−1.844 (−2.017 to −1.672)***	−0.026 (−0.232 to 0.181)	−0.025 (−0.232 to 0.182)
**Geographic residence (ref = urban)**			
Rural	−3.969 (−4.273 to −3.665)***	−1.541 (−1.798 to −1.284)***	−1.540 (−1.797 to −1.283)***
**Education (ref = no formal education)**			
Basic literacy or elementary school education		4.301 (4.118 to 4.485)***	4.302 (4.119 to 4.486)***
Middle or high school		7.072 (6.858 to 7.285)***	7.073 (6.859 to 7.286)***
Above		9.241 (8.675 to 9.086)***	9.242 (8.677 to 9.808)***
**The number of physical comorbidities (ref = 0)**			
1–2		−0.038 (−0.212 to 0.136)	−0.037 (−0.211 to 0.136)
≥ 3		0.143 (−0.036 to 0.321)	0.142 (−0.036 to 0.321)
**Feeling pain (ref = no)**			
Yes		−0.387 (−0.551 to 0.223)***	−0.387 (−0.551 to 0.224)***
**IADLs (ref = unimpaired)**			
Impaired		−0.825 (−1.021 to −0.629)***	−0.826 (−1.021 to −0.630)***
**Depressive symptoms (ref = no)**			
Yes		−0.665 (−0.817 to −0.513)***	−0.665 (−0.817 to −0.513)***
**Smoking (ref = no)**			
Yes		−0.121 (−0.315 to 0.073)	−0.120 (−0.315 to 0.074)
**Drinking (ref = no)**			
Yes		0.087 (−0.080 to 0.254)	0.087 (−0.080 to 0.254)
Body mass index		0.082 (0.063 to 0.100)***	0.082 (0.063 to 0.100)***
Household expenditure per capita (log)		0.254 (0.194 to 0.315)***	0.255 (0.194 to 0.315)***
Time * body function (ref = time * BF group 1)			
Time * BF group 2			−0.009 (−0.038 to 0.055)
Time * BF group 3			−0.029 (−0.169 to 0.111)
Time * BF group 4			−0.045 (−0.069 to −0.020)***
Time * BF group 5			0.019 (−0.038 to 0.076)
Time * BF group 6			−0.072 (−0.202 to 0.057)
**Random effects**			
**Level 2: Individual**			
Individual -variance	15.990***	9.316***	9.346***
**Level 1: Point in time**			
Point in time-variance	12.272***	12.273***	12.268***
−2*log-likelihood	247,955.6	243,591.2	243,575.6

(1) The number of observations in 2011, 2013, 2015, 2018, and 2020 were 10,149, 8,951, 8,967, 7,229, and 7,583, respectively. Figures in the table were parameter estimates based on unweighted multilevel models from 10,149 individuals consisting of 42,879 observations. (2) Model estimation was based on Maximum likelihood. (3) ref, the reference category; IADLs, instrumental activity of daily living.

(4) *P < 0.05, **P < 0.01, ***P < 0.001.

Table 3 presents the results of multilevel mediation models fit for Model 4. The model 4 revealed a significant indirect effect of BF on CF through SAP in group BF group 4 (β = −0.03, *P* < 0.01, mediation proportion: 4.32), while no such channeling effect of SAP was observed in other groups.

**TABLE 3:** Mediation analysis results of contribution of social activity participation using longitudinal data.

	Model 4			
	**BF → CF (total)** **Standardized coefficient (95% CI)**	**BF → CF (direct)** **Standardized coefficient (95% CI)**	**BF → CF (indirect)** **Standardized coefficient (95% CI)**	**Mediation proportion**
BF1	0.00 (Ref.)	0.00 (Ref.)	0.00 (Ref.)	n.a.
BF2	−0.29 (−0.60 to 0.01)	−0.23 (−0.53 to 0.06)	−0.06 (−0.09 to −0.03)***	n.a.
BF3	−0.87 (−1.73 to 0.03)	−0.77 (−1.64 to 0.13)	−0.10 (−0.18 to −0.01)*	n.a.
BF4	−0.67 (−0.83 to −0.51)***	−0.64 (−0.80 to −0.48)***	−0.03 (−0.05 to −0.01)***	4.32
BF5	−1.03 (−1.40 to −0.65)***	−1.00 (−1.37 to −0.62)***	−0.03 (−0.07 to 0.001)	n.a.
BF6	−1.63 (−2.39 to −0.90)***	−1.65 (−2.39 to −0.91)***	0.02 (−0.05 to 0.100)	n.a.

(1) Model 4: multilevel mediating model adjusting all covariates (age, geographic residence, education, physical comorbidities, feeling pain, IADLs, depressive symptoms, smoking, alcohol intake, BMI and household expenditure per capita, time) while regarding body function as exposure, social activity participation as mediator, and cognitive function as outcome. (2) Model estimation was based on Ordinary Least Squares of Maximum likelihood. (3) Ref, the reference category; BF means body function and the number after BF reflects the level of body function; CF, cognitive function decline; SAP, social activity participation; n.a., not applicable; CI, confidence interval. (4) “total” means “total effect,” “direct” means “direct effect,” “indirect” means “indirect effect.”

(5) *P < 0.05, **P < 0.01, ***P < 0.001.

## 4 Discussion

This longitudinal study aimed to investigate the associations between single/dual sensory impairment, poor physical performance, their combination and cognitive function in a sample of 10,149 Chinese participants aged 45 and above, using five-wave data from the China Health and Retirement Longitudinal Study (CHARLS). As mentioned in the background studies (Veronese et al., 2016; Stijntjes et al., 2017; Christensen et al., 2019; Schubert et al., 2019), the effects of sensory impairments and low physical performance on cognitive function have also been discussed separately in regions outside of China. Therefore, future research could enrich the findings of this study by exploring these effects in other populations.

For multilevel Models 1–3, individuals with low physical performance and combination of it with single/dual sensory impairment had poorer cognitive function after adjusting for potential covariates. This suggested that poor physical performance and sensory impairment were significant risk factors for cognitive decline, in line with previous studies (Veronese et al., 2016; Stijntjes et al., 2017; Christensen et al., 2019; Ikegami et al., 2019; Schubert et al., 2019; Liu et al., 2021; Zhao et al., 2021; Phillips et al., 2022). Although some results were not statistically significant, individuals with dual sensory impairments had poorer cognitive function than those with single sensory impairments, regardless of whether the subjects had low physical performance. This implied the impact of sensory impairments on cognitive function was probably cumulative. It is worth mentioning that this study explored the relationship between single/dual sensory disorders and cognitive function with the longest time span in China to date.

Moreover, our original first hypothesis posits that the combination of sensory impairment and low physical performance has a cumulative effect on cognitive decline. In comparison to individuals with only sensory impairment or poor physical performance, the results of point estimate in Model 1–3 indicating that the individuals with both indeed have worse performance of CF confirmed the cumulative negative effects of sensory disorders and low physical performance which was our first hypothesis. The cumulative effect refers to the statistical additive negative impact of sensory impairments and physical performance on cognitive function. In the descriptive analysis, the group with dual sensory impairment (regardless of whether accompanied by low physical performance) showed a high proportion of individuals with depressive symptoms. This suggests that dual sensory impairment (whether or not accompanied by low physical performance) may play a significant role in mental health, particularly regarding depressive symptoms. Since there is an association between depressive symptoms and cognitive function (Guan et al., 2022; Lu and Ruan, 2023), dual sensory impairment may independently affect cognitive dysfunction through depressive symptoms. These findings provide a new perspective on how dual sensory impairment influences cognitive function and highlight the importance of considering mental health factors in future interventions and research.

Meanwhile, we observed that individuals with only sensory impairment did not show a significantly worse CF while individuals with both sensory impairment and low physical performance showed. This may indicate that cognitive function was only affected when the physical performance of individuals with sensory impairments was affected. This inspired us to consider whether sensory impairment and poor physical performance coexist or occur separately when analyzing their impact on CF in the future.

Simultaneously, our study suggested that only individuals with low physical performance experienced not only performed worse on baseline CF but also had a faster rate of cognitive decline. This suggested that the adverse impact of poor physical performance on CF could persist for a long time. Therefore, whenever individuals experience poor physical performance, it is important to monitor for sustained cognitive decline and implement remedies measures such attending social activities. However, those with both low physical performance and single/dual sensory disorders did not experience a faster rate of cognitive decline. A previous study (Tu et al., 2020) identified three trajectories of cognitive decline based on cognitive function scores: slow decline, rapid decline, and stable function. The slow decline trajectory was linked to a low baseline cognitive function score (Tu et al., 2020). Therefore, we speculated that low physical performance combined with sensory impairment had an early and severe impact on cognition, but its effect on the rate of subsequent cognitive decline diminished after the occurrence of serious cognitive impairment. Other studies have also reached similar conclusions regarding the floor effect (Barbarotto et al., 2000; Ahn et al., 2024; Nitsch and Kalenscher, 2021). Future analyses may employ subgroup comparisons to investigate whether these baseline scores attain levels that are linked to reduced rates of cognitive decline. Moreover, it is worth mentioning that cognitive ability can generally be divided into fluid cognitive ability and crystallized cognitive ability. The latter is acquired through learning, training, and practice and does not decline with age; the former refers to the ability to solve new problems in novel environments, which declines with age and is more vulnerable to brain injury (Salthouse, 2006). Therefore, we speculate that sensory impairment and low physical performance may have a greater impact on fluid cognitive ability. However, this study did not include such detailed analyses, and future research could address this gap. Another possibility was that those severe physical damage had a sustained impact on the rate of cognitive decline. Individuals experiencing sustained cognitive decline may be unable to participate in the survey due to physical and cognitive limitations, and those who can participate may be survivors of severe physical damage.

Additionally, the results of this study, showing a potential mediating role of SAP in the association between low physical performance and CF, partially proved our second hypothesis. The mediating effect found in this study was not substantial, which may be due to the insufficient intensity and limited variety of SAP, potentially obscuring the true mediating effect. Previous studies have demonstrated a correlation between physical performance and depression (Tan et al., 2022), and between depression and cognition (Dehn and Beblo, 2019). Therefore, considering the participation in social activities can prevent depression (Wang et al., 2020), this could explain why the mediating effect of low physical performance on CF through SAP occurred. Therefore, older adults with sensory impairments and/or low physical performance can combat cognitive decline by improving their mental health status.

Moreover, SAP increased physical activity of individuals with a low physical performance. Increased physical activity has been shown to positively impact multiple physiological systems, including metabolic, skeletal, cardiovascular, and immune systems, among others, in older adults (Bangsbo et al., 2019). This may be due to chronic pathological mechanisms caused by low physical activity, such as inflammation, platelet aggregation, blood coagulation, and energy redistribution, which lead to changes in the cardiovascular system. These changes reduce the system’s ability to respond to environmental challenges, thereby diminishing the individual’s physiological reserve, accelerating the aging process, contributing to frailty, and ultimately resulting in a shortened healthy life expectancy (Charansonney, 2011).

Another hypothesis for the relatively low mediating effect of SAP is the potential reverse causality between cognitive function and SAP. For example, individuals with lower cognitive function may participate in fewer social activities, or there may be underlying confounding factors between cognitive function and SAP that obscure the mediating effect. Future studies could employ more advanced statistical methods to control for reverse causality and confounding factors, thereby obtaining a more accurate estimate of SAP’s mediating effect among these variables.

Consequently, participating in physical activity may still contribute to the improvement of CF for individuals with low physical performance. The government can implement more targeted interventions, such as organizing community activities like card games and chess that are suitable for individuals with lower levels of physical activity. Additionally, allocating more funding to conduct a wider variety of community activities across multiple regions could enhance accessibility. Moreover, while promoting social participation, the government should also focus on early screening and intervention for low physical performance and sensory impairments. This approach could facilitate primary prevention of cognitive impairment and secondary prevention of sensory impairments and low physical performance at a lower cost.

It is worth noting that the alpha value of the scale we used to measure social activity participation is not very high, which may impact the assessment of the mediating effect of social activity participation. Future research could explore scales with higher alpha values for social activity participation, such as revising or adding types of social activities, ensuring clarity for each indicator, and fully considering the dimensions of social activity participation to improve the accuracy of this type of research.

### 4.1 Strength and limitations

This study contributes to the existing literature in several ways. Firstly, when analyzing the impact of sensory impairment and poor physical performance on cognitive function, we considered whether they coexist or occur separately. Secondly, we employed a mediation approach to quantify the channeling influence of social activity participation on the cognitive decline which provided a practical preventive measure for the individuals with low physical performance or sensory impairment to mitigate the risk of cognitive impairment. Thirdly, we utilized a large-scale nationally representative longitudinal dataset spanning nine years, which minimized the potential bias of reverse causation and enhances the generalizability of our findings.

There are several limitations that should be acknowledged in this study. Firstly, due to the significant sample size differences in the BF group (such as BF3 and BF6), the resulting data imbalance may affect the reliability and accuracy of the outcomes. Additionally, due to the limitations of sample size and the consideration of data heterogeneity, as well as the fact that this study is exploratory in nature, we did not further categorize individuals with single impairments into auditory and visual impairments. This limits our ability to reflect the differences in their effects on cognitive function. Secondly, the reliance on self-reported measures for sensory impairment may introduce subjectivity and affect the accuracy of the results. It could be beneficial to consider more objective measures (e.g., hearing or vision tests) in future studies to enhance the accuracy of the findings. Thirdly, since samples with missing variables were directly excluded in this study, this may introduce selection bias. Fourthly, the longitudinal multilevel mediation proportion of SAP for individuals with low physical performance alone (4.32%) was relatively low, and for those with low physical performance combined with sensory impairment, it was not significant. These results suggested that there may be additional factors contributing to the complex relationship between sensory impairment, physical performance and cognitive decline.

## 5 Conclusion

This study indicated the cumulative effect of sensory impairments and low physical performance on cognitive function and implied that cognitive function was probably affected only when the physical performance of individuals with sensory impairments was compromised. Meanwhile, this study highlighted the mediating role of SAP between low physical performance and cognitive decline, providing insights into the underlying mechanisms linking physical performance and cognition. Hence, individuals with sensory impairment should take proactive action, such as participating more in hearing or vision protection training, to prevent low physical performance for protecting cognitive function. Besides, the government could formulate some policies for encouraging and promoting the organization of more social activities that individuals with low physical performance could participate in.

## Data Availability

The original contributions presented in this study are included in this article/Supplementary material, further inquiries can be directed to the corresponding author.
