# Supplementary material for: Contribution of social activity participation to the relationship between sensory impairment, physical performance and cognitive decline: a longitudinal study in China
Source: Front Aging Neurosci. 2024 Dec 11;16:1498354. doi: 10.3389/fnagi.2024.1498354 (PMC11668813; doi:10.3389/fnagi.2024.1498354)
Supplement: Supplementary file 1 [file Table_1.docx]

Supplementary Material

**Contribution of social activity participation to relationship between sensory disorders, physical performance and cognitive decline: A longitudinal study in China**

**Additional File**

Supplemental detailed information for variables

1.covariates

2. exposure variables

3. outcome variables

4. mediator

Supplementary tables and figures

Table S1. The questionnaire items of CESD-10 and its answer options and marks assigned

Table S2. Baseline characteristics between participants included and not included

**Supplemental detailed information for variables**

# covariates

## **Age**

Age was classified into the following four groups: 45-54, 55-64, and older than 65 years old.

## Geographic area

It was divided into rural or urban based on the National Bureau of Statistics of the People's Republic of China.

## Education

In the original questionnaire of CHARLS, the answers for education were divided into eleven categories, including 1) no formal education (illiterate); 2) did not finish primary school but can read; 3) Sishu (private tutoring); 4) elementary/primary school; 5) middle school; 6) high school; 7) vocational school; 8) two-/three-year college/associate degree; 9) four-year college/Bachelor’s degree; 10) Master’s degree; 11) Doctoral degree/Ph.D. Due to the relatively low education level of older people in China (thirty-three per cent of participants had an educational level higher than primary school).

we classified education level into four groups:

answer 1) -- no formal education;

answer 2) to answer 4) --- basic literacy or elementary school education,

answer 5) to answer 7) --- middle or high school;

answer 8) to answer 11) ---above.

## Physical comorbidity

The scope of physical comorbidity in this study contained hypertension, dyslipidemia, diabetes or high blood sugar, cancer or malignant tumor, chronic lung disease, liver disease, heart disease, stroke, kidney disease, stomach or digestive disease, arthritis or rheumatism, and asthma. The number of physical comorbidities was calculated and categorized as 0,1-2 and ≥ 3.

## Feeling pain

Feeling pain was self-reported via a question: “Are you often troubled with any body pains?”.

## IADLs ( instrumental activity of daily living)

IADLs were evaluated through doing housework, cooking, taking medicine, shopping, and taking care of finances. Participants who reported having any difficulty in any items were classified as with IADLs impaired.

## Depression

It was measured by Chinese version of 10-item Center for Epidemiologic Studies Depression (CESD-10) Scale which reflects the respondents’ depressive symptoms experienced over the previous week. The answers for each item included “rarely (< 1 day/week)”; “some days (1–2 days/week)”; “occasionally (3–4 days/week)”; and “most (5–7 days/week)”. Each item was scored according to their frequency of symptoms. The questionnaire items for the CESD-10 and their answer options and score allocations are shown in **Table S1.**

## **Smoking**

It was self-reported via a question: “Have you ever chewed tobacco, smoked a pipe, smoked self-rolled cigarettes, or smoked cigarettes/cigars?”.

## Drinking

It was self-reported via a question: “Did you drink any alcoholic beverages, such as beer, wine, or liquor in the past year? How often?”. The answers for this question included three: 1)Drink more than once a month; 2) Drink but less than once a month; 3) None of these. And we considered answer 1) and 2) as “yes”, and answer 3) as “no”.

## BMI

It was calculated from self-reported weight in kilograms divided by the square of height in meters (kg/m2).

## Average annual household expenditure per capita

Average annual household expenditure per capita was calculated by taking total household consumption divided by the number of people in the household. Total household consumption comprised of a wide range of expenditures, which was the sum of food consumption and non-food consumption. To capture the non-linear relationship between income and outcome variables, the average annual household expenditure was log-transformed in the analysis.

# exposure variables

## physical performance

It was measured by short physical performance battery (SPPB) included standing balance test, the gait speed test and the five-time chair stand test. A summary physical performance score was calculating the sum scores of three tests above ranging from 0-12 points with 4 points for each test.

**1)** For balance test, participants attempted to maintain three positions for 10 seconds each: (a) a side‐by‐side position; (b) semi-tandem stand (the heel of one foot beside the big toe of the other foot); and (c) tandem position (the heel of one foot in front of and touching the toes of the other foot).

The individuals were score 4 if they could hold tandem position for full 10 seconds without stepping out of place or grabbing hold of anything; individuals were scored 3 if they could hold tandem position for 3~9 seconds; individuals were scored 2 if they could hold tandem position for under 3 seconds and hold semi-tandem stand for full 10 seconds; individuals were scored 1 if they could hold tandem position for under 10 seconds and hold side‐by‐side position for full 10 seconds; individuals were scored 0 if they could only hold side‐by‐side position for under 10 seconds.

**2)** For Gait speed test, participants were scored according to the faster time of walking a 2.5m distance at a normal pace.

The participants were scored 4 if their walking time were <=3.19; participants were scored 3 if their walking time were 3.2 to 4.0 seconds; participants were scored 2 if were 4.1 to 5.6 seconds; participants were scored 1 if their walking time were ≥ 5.7 seconds.

**3)** For five-time chair stand test, the score was defined by the amount of time needed for participants to rise continuously five times from a 47‐cm chair with folding arms across their chest.

Quartiles of performance for the repeat chair stands were used to define scores as follows: score of 0:unable to finish this test; score of 1: > 16.7 seconds; score of 2: 16.6–13.7 seconds; score of 3: 13.6–11.2 seconds; score of 4: ≤ 11.1 seconds.

## sensory disorders

Sensory impairment assessed by investigating, “Do you have vision or/and hearing problems?” we defined single sensory impairment as having problems on vision or hearing and duel sensory impairment as having problems on both vision and hearing, otherwise no sensory impairment.

# **outcome variable**

Cognitive function (CF) was assessed using a methodology adapted from the American Health and Retirement Study (HRS), focusing on four dimensions: (1) orientation, (2) memory, (3) computation, and (4) drawing.

1) Orientation: The orientation dimension involved questions that asked individuals to identify year, month, day, day of the week, and current season. A total score of five points was assigned to the orientation dimension, with one point given for each correct response.

2) Memory: Memory assessment included the presentation of a 10-words list, and immediate recall was assessed by counting the number of words participants could remember immediately after the presentation. Additionally, delayed recall was assessed by recording the number of words individuals could remember after a 5 minutes interval [30]. The total score of memory dimension was calculated by summing scores of immediate and delayed word recall, with a maximum score of 20 points (one point per correct recalled word).

3) Computation: Computation was evaluated by performing successive subtraction of 7 from 100 for five, with one point awarded for each accurate calculation.

4) Drawing: The drawing dimension involved participants replicating a displayed image of two overlapping pentacle stars, with one point awarded for successfully reproducing the drawing.

The overall cognitive function score was determined by summing the scores from the four dimensions: orientation (5 points), computation (5 points), memory (20 points), and drawing (1 point), resulting in a total score ranging from 0 to 31. A higher score on the CF test indicates a better CF.

# mediator

Social activity participation (SAP) in the last month was regarded as mediator. The CHARLS questionnaire included six categories of social activities, including: (1) Interacted with friends; (2) Played Ma-jong, played chess, played cards, or went to community club; (3) Provided help to family, friends, or neighbors who do not live with you; (4) Went to a sport, social, or other kind of club; (5) Took part in a community-related organization; (6) Done voluntary or charity work. For each social activity, there were four responses for the participants including: (1) None; (2) Not regularly; (3) Almost every week; (4) Almost daily.

We assigned possible responses as: None = 0, Not regularly/ Almost every week = 1, and Almost daily = 2. And the level of SAP was measured by summing up the responses for each of the six activities. The total scores of SAP ranged from 0 to 12, with the higher scores denoting the higher levels of SAP.

**Supplementary tables and figures**

**Table S1. The questionnaire items of CESD-10 and its answer options and marks assigned**

| Questionnaire items | Answer options and marks assigned | | | |
| --- | --- | --- | --- | --- |
| I was bothered by things that don’t usually bother me | 0=less 1 day | 1= 1-2 days | 2= 3-4 days | 3= 5-7 days |
| I had trouble keeping my mind on what I was doing | 0=less 1 day | 1= 1-2 days | 2= 3-4 days | 3= 5-7 days |
| I felt depressed | 0=less 1 day | 1= 1-2 days | 2= 3-4 days | 3= 5-7 days |
| I felt everything I did was an effort | 0=less 1 day | 1= 1-2 days | 2= 3-4 days | 3= 5-7 days |
| I felt hopeful about the future | 0=5-7 days | 1= 3-4 days | 2= 1-2 days | 3= less 1 day |
| I felt fearful | 0=less 1 day | 1= 1-2 days | 2= 3-4 days | 3= 5-7 days |
| My sleep was restless | 0=less 1 day | 1= 1-2 days | 2= 3-4 days | 3= 5-7 days |
| I was happy | 0=5-7 days | 1= 3-4 days | 2= 1-2 days | 3= less 1 day |
| I felt lonely | 0=less 1 day | 1= 1-2 days | 2= 3-4 days | 3= 5-7 days |
| I could not get “going” | 0=less 1 day | 1= 1-2 days | 2= 3-4 days | 3= 5-7 days |

**Table S2. Baseline characteristics between participants included and not included**

| Characteristics | Included (n=10149) | Excluded (n=2721) | *P* value |
| --- | --- | --- | --- |
| **Cognitive function,mean ± SD** | 14.60±5.52 | 9.56±6.64 | <0.001 |
| **Body function level, n (%)** |  |  | <0.001 |
| BF group 1 | 6282 (61.9) | 1323 (48.7) |  |
| BF group 2 | 572 (5.6) | 150 (5.5) |  |
| BF group 3 | 64 (0.6) | 30 (1.1) |  |
| BF group 4 | 2717 (26.8) | 892 (32.8) |  |
| BF group 5 | 420 (4.1) | 254 (9.3) |  |
| BF group 6 | 94 (0.9) | 70 (2.6) |  |
| **Cognitive function,**  **mean ± SD** | 14.60±5.52 | 9.56±6.64 | <0.001 |
| **Social activity participation，mean ± SD** | 1.01±1.24 | 0.85±1.14 | <0.001 |
| **Age (years at baseline), mean ± SD** | 58.34±8.80 | 60.13±11.83 | <0.001 |
| **BMI， mean ± SD** | 23.61±3.93 | 23.06±3.86 | <0.001 |
| **Gender, n (%)** |  |  | <0.001 |
| Male | 4885 (48.1) | 1167 (43.0) |  |
| Female | 5264 (51.9) | 1545 (57.0) |  |
| **Geographic area, n (%)** |  |  | 0.536 |
| Rural | 878 (8.7) | 225 (8.3) |  |
| Urban | 9271 (91.3) | 2494 (91.7) |  |
| **Education, n (%)** |  |  | <0.001 |
| No formal education | 2537 (25.0) | 964 (35.5) |  |
| Basic literacy or elementary school education | 4227 (41.6) | 1081 (39.8) |  |
| Middle or high school | 3207 (31.6) | 633 (23.3) |  |
| Above | 178 (1.8) | 41 (1.5) |  |
| **The number of physical comorbidities, n (%)** |  |  | <0.001 |
| 0 | 3396 (33.5) | 808 (29.7) |  |
| 1-2 | 3102 (30.6) | 811 (29.8) |  |
| ≥3 | 3651 (36.0) | 1102 (40.5) |  |
| **Feeling pain, n (%)** |  |  | <0.001 |
| No | 6914 (68.1) | 1701 (62.7) |  |
| Yes | 3235 (31.9) | 1012 (37.3) |  |
| **IADLs, n (%)** |  |  | <0.001 |
| No | 8452 (83.3) | 1934 (71.1) |  |
| Yes | 1697 (16.7) | 786 (28.9) |  |
| **Depressive symptoms, n (%)** |  |  | <0.001 |
| No | 6240 (61.5) | 1420 (52.2) |  |
| Yes | 3909 (38.5) | 1301 (47.8) |  |
| **Smoke, n (%)** |  |  | 0.078 |
| No | 6121 (60.3) | 1693 (62.2) |  |
| Yes | 4028 (39.7) | 1027 (37.8) |  |
| **Drink, n (%)** |  |  | <0.001 |
| No | 6701 (66.0) | 1932 (71.0) |  |
| Yes | 3448 (34.0) | 789 (29.0) |  |
| **Household expenditure per capita(log), mean ± SD** | 7.31±1.19 | 7.22±1.23 | <0.001 |

Note: (1) All the variable were measured at baseline interview. (2) The *P-*value in continuous variables were tested using one-way analysis of variance (ANOVA) and in categorical variables using chi-square tests. (3) SD = standard deviation, BMI = body mass index, IADLs = instrumental activity of daily living, BF = body function (Body function groups were classified by whether individuals suffered from sensory disorders or poor physical performance which mentioned in Method of this study.)
